# Nurses’ research utilization two years after graduation—a national survey of associated individual, organizational, and educational factors

**DOI:** 10.1186/1748-5908-7-46

**Published:** 2012-05-18

**Authors:** Henrietta Forsman, Ann Rudman, Petter Gustavsson, Anna Ehrenberg, Lars Wallin

**Affiliations:** 1 Dalarna University, School of Health and Social Studies, Falun, Sweden; 2 Karolinska Institutet, Department of Neurobiology, Care Sciences and Society, Stockholm, Sweden; 3 Karolinska Institutet, Department of Clinical Neuroscience, Stockholm, Sweden; 4 Karolinska University Hospital, CRU (Clinical Research Utilization), Stockholm, Sweden

## Abstract

**Background:**

Nurses’ research utilization (RU) as part of evidence-based practice is strongly emphasized in today’s nursing education and clinical practice. The primary aim of RU is to provide high-quality nursing care to patients. Data on newly graduated nurses’ RU are scarce, but a predominance of low use has been reported in recent studies. Factors associated with nurses’ RU have previously been identified among individual and organizational/contextual factors, but there is a lack of knowledge about how these factors, including educational ones, interact with each other and with RU, particularly in nurses during the first years after graduation. The purpose of this study was therefore to identify factors that predict the probability for low RU among registered nurses two years after graduation.

**Methods:**

Data were collected as part of the LANE study (Longitudinal Analysis of Nursing Education), a Swedish national survey of nursing students and registered nurses. Data on nurses’ instrumental, conceptual, and persuasive RU were collected two years after graduation (2007, n = 845), together with data on work contextual factors. Data on individual and educational factors were collected in the first year (2002) and last term of education (2004). Guided by an analytic schedule, bivariate analyses, followed by logistic regression modeling, were applied.

**Results:**

Of the variables associated with RU in the bivariate analyses, six were found to be significantly related to low RU in the final logistic regression model: work in the psychiatric setting, role ambiguity, sufficient staffing, low work challenge, being male, and low student activity.

**Conclusions:**

A number of factors associated with nurses’ low extent of RU two years postgraduation were found, most of them potentially modifiable. These findings illustrate the multitude of factors related to low RU extent and take their interrelationships into account. This knowledge might serve as useful input in planning future studies aiming to improve nurses’, specifically newly graduated nurses’, RU.

## Background

Research utilization (RU) in newly graduated registered nurses has been identified as being conspicuously low in previous population-based longitudinal studies, where about 50% of the nurses one, two, and three years after graduation rated their use of research in clinical practice as low or very low [1,2]. Further, the results indicated that low users tended to become even lower over time between the first and second year after graduation [2]. After various educational reforms, evidence-based nursing and RU are strongly emphasized internationally in nursing education. A number of challenges remain, however, regarding the content of nursing education and the transition from education into working life (e.g., the integration of education and practice as well as the ability of students to access and interpret evidence) [3,4].

RU, per definition, is the use of research evidence exclusively. Research evidence constitutes a subset of evidence-based practice (EBP), which also includes the use of nonresearch sources of evidence (e.g., clinical experience) [5]. Consequently, the term EBP is, in this present study, treated as a concept closely related to, and as including, the use of research-based knowledge in practice. In 1992, evidence-based medicine appeared as an emerging paradigm for medical practice [6] and has since then spread among other healthcare professions. EBP aims at high-quality, safe, and cost-effective care based on the best available knowledge [7]. However, the implementation and subsequent use of evidence-based knowledge in clinical practice are far from straightforward and have recurrently been reported as a difficult undertaking in medicine [8], physiotherapy [9], occupational therapy [10], and nursing [11,12]. According to a recent systematic review of the extent of nurses’ RU in clinical practice, nurses reported their RU to an extent designated as moderate-high in the majority of the included studies [13]. The lack of standard measures for RU, however, makes it difficult to compare findings across studies. In nursing, barriers to RU have been studied and found to originate from the organization, the evidence itself, and the nurse as an individual [14]. This illustrates that RU in clinical practice is a complex phenomenon where many factors operate on many levels. A major challenge is to gain a better understanding of factors that facilitate and hinder the use of research-based knowledge in practice, including individual and organizational factors.

### Individual characteristics

In his classical work on diffusion of innovations, Rogers [15] describes how individuals differ in their adoption of new ideas/innovations. Individuals’ adoption behaviors differ based on their innovativeness or time to adoption. A recent systematic review of individual determinants of RU provides support for a positive association between RU and nurses’ positive attitudes toward research, nurses’ conference attendance and/or attendance at in-service training, having a graduate degree, current role, clinical specialty, and job satisfaction [16]. In the BARRIERS scale, commonly used for assessing barriers to RU, examples of barriers related to the nurse are feelings of not being capable to appraise research, not seeing the value of translating research into practice, and being unwilling to change or try new ideas [17]. However, a focus on the use of evidence solely as an individual activity, in which the nurse is seen as a “rational agent” able to search, appraise, and implement evidence in practice, has been criticized for its underlying assumptions of rationality and linearity [18]. Since the individual nurse does not work in isolation, the influence of multiple factors, especially contextual ones, needs to be recognized as well [18].

### Organization and context

**Figure 1:**
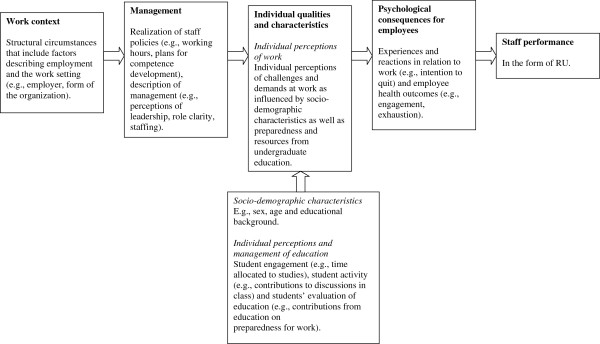
The analytic schedule with its elements, sub-elements and their operationalizations.

The relationship between RU and factors related to the organizational context has been investigated in several studies [19-21]. Barriers to RU related to the organization include inadequate facilities for implementation, lack of time to read research, and insufficient time on the job for implementation of new ideas [14]. In the PARiHS framework (Promoting Action on Research Implementation in Health Services), context is viewed as an important element for research implementation, focusing on culture, leadership, and evaluation [22,23]. Several studies support the importance of these three elements (i.e., a “positive” context) for RU to take place [19,21,24]. However, which components of context that actually exercise an effect on nurses’ use of research are not always clear. A common argument is that factors related to the organizational context need to be further studied. In addition, according to Dopson et al. [25], context exercises its influences on different layers in the organization, such as the outer government health policy layer, the inner regional/local government layer, and finally, single organization and individual practitioner layers. Each layer has to be examined for its specific influences on evidence implementation because influences from the different layers are complex and can be seen in a variety of combinations [25]. An example of modelling these layers (in this study, denominated as *levels*) and the individual in the organization is the National Health Service (NHS) model used for the NHS staff survey [26]. The NHS model is “an architecture for understanding the links between the context of work, management of people practices, psychological consequences for staff, staff behaviour and performance, and employee health, performance and patient care in the NHS” [27], p. 1]. An adapted version of the NHS model was used as an analytic schedule in this present study (Figure 1).

### Nursing education

Translating nursing education level into university level has involved problems in Sweden as well as internationally. Difficulties include the gap between theory and practice, as well as a mis-fit of new graduates to the practice setting relating to the tension between academic demands and clinical skills [28,29]. In Sweden, several nursing schools have been criticized for not reaching learning goals adequate for the academic level of the education [30]. Educational outcomes focusing on skills necessary to perform research-based care are, to our knowledge, poorly studied. However, students’ self-assessed engagement and benefits from education were assessed in two cohorts of graduating Swedish nursing students [31,32]. The vast majority of the students (≥80%) reported that they had developed their ability to think critically and analytically, independently seek knowledge, and take responsibility for their own knowledge development during their education. Furthermore, in one of the cohorts [31], 86% of the students rated that the education contributed to their ability to appraise research findings. Students’ assessment of their learning outcomes and benefits from undergraduate education in relation to their subsequent clinical behavior was not studied, however.

Studies on newly graduated nurses’ RU are rare. However, Swedish nurses have reported their application of RU [1,2] as relatively low the first years postgraduation, where about half of the national samples reported that they never or only occasionally used research. These findings raise the question of whether skills gained during education (assuming that the skills actually have been obtained during education) have effects on nursing practice (e.g., on nurses’ ability to use research to inform practice). To our knowledge, educational factors that may be important for nurses’ subsequent use of research in clinical practice have not been previously investigated and were therefore included in this study.

To sum up, factors associated with RU have been investigated in numerous previous studies and factors have, in most cases, been categorized as either individual or organizational/contextual. Traditionally, the focus has been on individual factors, but there is an increased focus on organizational factors [18]. However, both individual and organizational factors have been insufficiently studied [16,19]. In addition, their interrelationships and associations to RU are complex and their influence is exerted at different levels [25]. There is a lack of studies that take this complexity into consideration and that include newly graduated nurses. Furthermore, it should be noted that definitions of variables as being either individual or organizational are not clear-cut. Some variables appear both in reviews on individual determinants [16] and on contextual factors [19], illustrating their overlapping nature. Despite the fact that the transition of nursing education into higher education has been described as troublesome in many ways [28-30], studies on educational factors in relation to subsequent clinical performance (e.g., RU) are lacking. Because of previous findings of extensively low RU among newly graduated nurses, the focus for further analyses was directed toward identifying factors related to low extent of RU, rather than factors associated with high use that have been commonly examined in other studies.

### Aim

The aim of this study was to identify factors that predict the probability for low RU among registered nurses two years after graduation.

## Methods

### Design

The present study was part of the Swedish LANE project (Longitudinal Analysis of Nursing Education [33]). LANE is a Swedish national survey with a prospective design in which data on individual characteristics as well as educational and work contextual conditions have been collected from 2002 until 2010 among nursing students and registered nurses. Data for this study were collected in the cohort comprising students graduating in 2004. In comparisons of cohort representativeness with population data, no differences were found on a number of demographic variables tested [33].

### Participants

**Figure 2:**
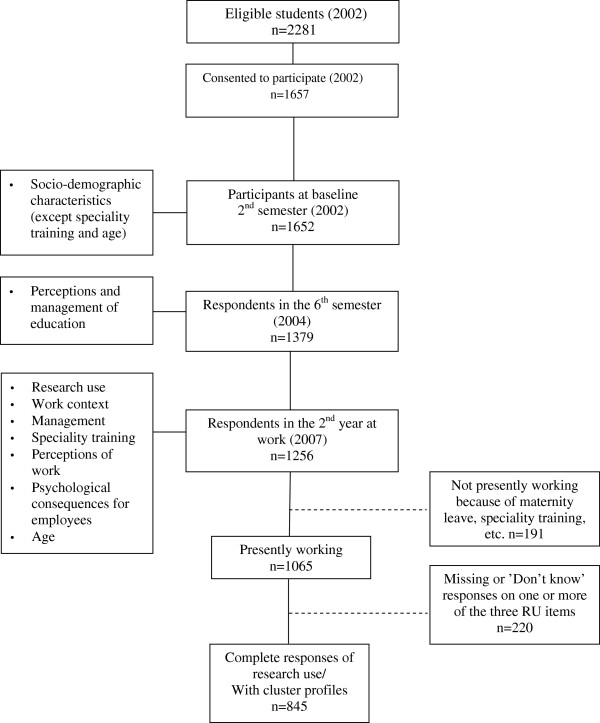
Overview of data collection, sample size and variables as measured at different time points.

An overview of data collection, sample size, and variables at the different time points is presented in Figure 2. Nursing students were recruited in 2002 from 24 of Sweden’s 26 nursing schools, where nursing education corresponds to a three-year university program, resulting in a Bachelor of Science in Nursing. The sample for this study was taken from the fifth data collection (i.e., year 2007, when participants had been registered nurses for two years). The number of respondents for that survey wave was 1,256. Of those nurses, 1,065 were working in the healthcare sector at the time of data collection and thus were eligible for our study. Data on individual characteristics, including individual perceptions and management of education, were gathered from previous data collections (second and sixth term of education, in 2002 and 2004, respectively). Individuals with missing values or those who responded “don’t know” in the outcome variable, RU, were excluded, as were those individuals not working as nurses at the time of data collection. Thus, the final sample for this study was 845 nurses (Figure 2).

Differences between the sample (n = 845) and excluded individuals (Figure 2) were tested using a number of variables related to demographic characteristics, health, and previous experience of healthcare work. The only difference found was that respondents included in the sample more often had already had children at baseline. This finding was not surprising since maternity leave is a major reason for presently not working (and thereby not being included in our sample) [33], and probably also more common among respondents who did not have children at baseline.

### Data collection

#### Research utilization

Data on RU were collected using three single items representing instrumental (direct), conceptual (indirect), and persuasive (symbolic) RU [2,34,35]. Instrumental RU represents a concrete application of research findings in patient care, conceptual RU corresponds to enlightening use to change an attitude or a way of thinking, and persuasive RU is when research evidence is used to influence others [2,34,35]. The conceptualization of the three different kinds of RU derive from social science and the work by Rich [36,37] and Weiss [38] (instrumental and conceptual RU, respectively) and from Pelz [39] and Beyer & Trice [40] (symbolic/persuasive RU). The measures used were originally developed by Estabrooks [34,35]. According to a recent systematic review on instruments for RU measurement [41], Estabrooks’s instrument has repeatedly shown validity evidence related to content as well as the response process and in relation to other variables. Explicit RU definitions, consistent through study reports, constitute a strength of this instrument [41], and the typological structure of RU was appropriate for our study aim. The measures have been translated and adapted for use in the Swedish context, in which the validity and reliability of the items have been supported [1,2]. For each RU item, a definition of RU was presented, followed by three examples of that kind of RU. Respondents were asked to rate the extent of their RU during the past four working weeks on a scale from 1 to 5: 1 = never, 2 = on some shifts, 3 = on about half of the working shifts, 4 = on more than half of the working shifts, 5 = on almost every shift. A “don’t know” alternative was also available.

#### Individual, educational, and organizational/contextual variables

Several of the factors included in this study have previously been used for assessing their relationships to RU or evidence-based care (e.g., staffing/workload [20,24], burnout/emotional exhaustion [24,42], age, clinical specialty, working full- or part-time [43], role clarity [22], and leadership [22,42,44]). In this present study, scales used to measure organizational factors, individual perceptions of work, and psychological consequences for employees were mainly taken from the QPS_Nordic_ (General Nordic Questionnaire for Psychological and Social Factors at Work) [45], composed to measure psychological, social, and organizational work conditions based on theories and models of organizational behavior, work motivation, job satisfaction, job stress, well-being, and health. The QPS_Nordic_ has been thoroughly evaluated in the Nordic countries and has shown good psychometric properties across occupational groups [46,47]. The scales measuring disengagement and exhaustion correspond to the two core dimensions of burnout according to the Oldenburg Burnout Inventory [48]. The scales have been translated into Swedish and also back-translated, and previous studies support the validity for the Swedish version among healthcare workers [49]. Some of the variables measuring perceptions and management of education originated from the National Survey of Student Engagement (NSSE) [50,51] and the Swedish corresponding survey (*Studentspegeln*/*the ‘Student Mirror’*) [52]. Psychometric properties of variables included in the Student Mirror have been assessed by an expert group at the Swedish National Agency for Higher Education [52]. Some variables used in this study were developed specifically for use in the LANE study, and the content of the LANE questionnaire has been reviewed at the technical and language laboratory at Statistics Sweden [33]. See Additional file 1 for a list of all variables and their origin.

An analytic schedule, consisting of elements and subelements, to select and organize the variables was developed from the NHS model, as presented by Michie and West [27]. The model was originally used to guide the preparation of the annual NHS staff survey of work conditions and health among healthcare employees in the United Kingdom. The NHS model is based on previous research [27] and was adapted for use in the present study. Originally, the outcome in the model was organizational performance in the form of patient outcomes. While the layered structure of the model has been retained, our adaptation mainly concerned the outcome (here, employee behavior, specifically nurses’ RU), and we included individual characteristics, such as individual perceptions and management of education, to fit the purpose of our study. The analytic schedule differentiates levels in the organization and the individual’s position in the organization. Figure 1 presents an illustration of our hypotheses with respect to the interrelationships between elements, subelements, and the outcome, as well as for viewing the variables included in each element/subelement. The levels are illustrated by the elements: Work Context and Management (representing organizational factors) and individual factors represented by Individual Qualities and Characteristics (including the three subelements Individual Perceptions of Work, Sociodemographic Characteristics, and Individual Perceptions and Management of Education) and Psychological Consequences for Employees. Staff Performance constitutes the outcome, RU. The schedule entails a ‘quasi-causality’ approach (i.e., it illustrates a process by which elements and subelements are hypothesized to primarily influence each other in the direction from context to performance, but where the opposite directions of associations, and also feedback loops, are conceivable). The underlying assumptions of the schedule were as follows: the overall work context is assumed to influence management within the context. Management, together with sociodemographic characteristics and individual perceptions and management of education, is expected to influence individuals’ perceptions of their work. Taken together, this is assumed to result in psychological consequences at work for the employee and, ultimately, in the final outcome, staff performance (in the form of RU).

### Ethical considerations

The respondents gave their informed consent to participate, and their confidentiality was protected. The study was approved by The Regional Research Ethics Committee at Karolinska Institutet, Stockholm (Dnr 01-045) and the Regional Ethical Review Board in Stockholm (Dnr 04-587).

### Data analysis

#### Research utilization

In our previous study [2], seven patterns of RU were found in the new graduates two years after graduation. The patterns were composed of individual response profiles based on each participant’s assessment of the three RU items. A profile thereby illustrated the individual’s overall RU behavior rather than the application of each kind of RU separately, constituting a composite measure of RU. The outcome variable in this study was created by using a cut-off separating individuals with overall low RU on all three items (i.e., subgroups of individuals rating on average 2.3 or lower on each of the RU items on the response scale ranging from 1 to 5, n = 464, 55%) from the rest (i.e., subgroups of individuals rating higher RU in one or more of the three RU kinds n = 381, 45%) [2].

#### Individual, educational, and organizational/contextual variables

Independent variables were mainly dichotomized (cut-offs are presented in detail in Additional file 1 in the column “Response categories”). Some variables were measured using multi-item instruments (in those cases, the number of items are indicated in Additional file 1). For those variables, ratings on the items were summarized and mean values calculated for each person.

#### Logistic regression modelling

To identify factors related to low RU, a three-step regression procedure was applied. All regression analyses were performed in SPSS version 16.0 (SPSS Inc., Chicago, IL, USA). Results, including odds ratios (ORs) and confidence intervals (CIs), *p* < .05, are presented in Table 1. The analyses were performed according to the steps below:

• Step 1: Bivariate logistic regression. Since relatively little is known about the relationship between low RU and the variables included in this study, the associations between RU and the variables were first analysed by means of bivariate logistic regression for a descriptive purpose.

• Step 2: Selection of variables to be included in the final logistic regression model. The analytic schedule (described above and in Figure 1) was used to organize the variables in a regression model. Taking multicollinearity between the independent variables into account, variables for the final logistic regression model were selected in this step based on separate regression models for each element/subelement in the analytic schedule, including all variables belonging to the specific element. Variables that showed significant relationships with RU were included in the final model.

• Step 3: The final logistic regression model. Variables from step 2 were entered sequentially, one element/subelement at a time, creating the final regression model (n = 736). The contribution from each element/subelement was tested by delta chi-square values together with additional model evaluation statistics (−2 log likelihood and Hosmer-Lemeshow test).

**Table 1:** Results from the logistic regression analyses, Step 1 (bivariate analyses) and Step 3 (final model)

**Independent variables**	**OR (CI) for low RU Step 1**	***p***	**OR (CI) for low RU Step 3**	***p***
***Work context***				
Permanent employment position	1.13 (0.86-1.49)	.379		
Clinical setting				
Hospital care (acute somatic care)	ref		ref	
Primary care (community health centers, home care)	(0.67-2.71)	.409	1.27 (0.59-2.76)	.540
Elder care (special housing for seniors)	1.08 (0.71-1.64)	.725	1.25 (0.79-1.96)	.345
Psychiatric care (hospitals and outpatient clinics)	3.65 (2.10-6.37)	.000	3.67 (1.92-7.03)	.000
Work >75% of full-time	1.07 (0.74-1.55)	.729		
Work shifts				
Day, evening, night	ref			
Monday to Friday (day, evening)	1.35 (0.88-2.07)	.169		
Night	0.87 (0.52-1.45)	.593		
***Management***				
Work overtime several times per week	0.70 (0.50-0.97)	.033		
Adequate staffing compared with patients’ needs of care	1.30 (0.98-1.73)	.067	1.43 (1.03-1.98)	.031
No/unknown individual plan for competence development	1.40 (1.05-1.87)	.020		
Role ambiguity	1.63 (1.19-2.22)	.002	1.44 (1.00-2.06)	.050
Deficient leadership	1.33 (0.99-1.79)	.063		
***Individual qualities and characteristics***				
*Sociodemographic characteristics*^a^				
Men	2.10 (1.31-3.36)	.002	1.88 (1.10-3.23)	.021
Age >30 years	1.08 (0.82-1.42)	.570		
No previous assistant nurse training	1.03 (0.79-1.36)	.824		
Further study after nursing degree	1.60 (1.09-2.35)	.017	1.40 (0.88-2.21)	.152
*Individual perceptions and management of education*^a^				
Low global importance of studies	1.52 (1.10-2.11)	.011		
Time allocated to studies:				
Full-time	ref			
>Full-time	0.68 (0.48-0.96)	.030		
≤75% of full-time	1.17 (0.83-1.64)	.381		
Low student activity: asking questions in class	1.33 (0.94-1.90)	.108		
Low student activity: contribution to discussions in class	1.57 (1.13-2.19)	.008	1.66 (1.16-2.39)	.006
Low educational quality (scientific theory and method)	1.10 (0.81-1.48)	.550		
Feel unprepared to manage work as nurse	1.42 (1.06-1.91)	.020		
*Individual perceptions of work, second year*				
High job demands	0.90 (0.67-1.20)	.476		
Low challenge at work	2.30 (1.57-3.37)	.000	2.03 (1.31-3.15)	.002
Low control at work	0.86 (0.51-1.43)	.560		
***Psychological consequences for employees***				
Often think about leaving the profession	1.53 (0.99-2.37)	.057		
High disengagement	1.57 (1.04-2.37)	.033		
High exhaustion	1.08 (0.81-1.44)	.604		
Low mastery at work	1.48 (1.10-1.99)	.010	1.29 (0.91-1.83)	.149

OR = odds ratio; CI = confidence interval.

Bold headings in italics = elements. Headings in italics = subelements. Results represent 95% CIs.

^a^Sociodemographic Characteristics and Individual Perceptions and Management of Education were entered together as one block in the model based on our hypothesis that both of these two blocks influence Individual Perceptions of Work in the second year.

## Results

Results from bivariate analyses (step 1) and the final logistic regression model (step 3) are shown in Table 1. Variables resulting from the element/subelement specific regression models (step 2 analyses) correspond to the ones included in the final model. Here, we focus on the result of the final regression model.

All elements and subelements, except for the element representing Psychological Consequences for Employees, significantly contributed to the explanation of the outcome, low RU (see Additional file 2 for model evaluation statistics and Table 1 for ORs). Representing Work Context, clinical setting was significantly associated with RU, that is, nurses working in psychiatric care were more likely to be low research users than those working in hospital care (OR = 3.67). Within the Management element, nurses experiencing adequate staffing in relation to patients’ needs of care (OR = 1.43), as well as those perceiving role ambiguity (OR = 1.44), were more likely to be low research users. Among the variables representing Individual Perceptions of Work, nurses who did not experience work as a positive challenge were more likely to be low users of research (OR = 2.03). Representing Individual Qualities and Characteristics, being male was associated with low RU (OR = 1.88) as well as low student activity (discussion in class) during undergraduate education (OR = 1.66).

## Discussion

This study provides new knowledge about the factors associated with low use of research among nurses. It focuses on new graduates—a group that has only rarely been studied in research on these issues. Furthermore, our sample is homogeneous in that it includes nurses who graduated from university at the same time (i.e., 2004). As a result of the multivariate approach, several of the variables that showed significant associations to the outcome in bivariate analyses proved nonsignificant in the end, which was due to their shared variance with other variables. Individual factors, including perceptions and management of education, and variables from different levels of the organization were identified as associated with low RU, illustrating the multifarious influences on RU.

Starting out from the outcome (i.e., low RU) and moving from right to left in our proposed analytic schedule, we can conclude that the element Psychological Consequences for Employees was not associated with RU in the final regression model. Included in that element were emotional exhaustion and disengagement, the two core dimensions of burnout according to the Oldenburg Burnout Inventory [48]. Several studies have found that emotional exhaustion has a negative effect on nurses’ commitment to caring and work environment improvement [42] and individual behavior change in relation to EBP [53] and RU [24]. In the present study, disengagement showed a significant association to low RU in the bivariate analysis, but did not remain significant in the final regression model; the dimension of emotional exhaustion, on the other hand, was nonsignificant in all analyses.

Continuing to move from right to left in our analytic schedule, Individual Perceptions of Work is the next subelement, where we found that individuals who experienced work as less challenging were more likely to be low research users. According to the schedule, management influences the perception of challenge at work. Pertaining to the Management element, staffing showed a rather unexpected association to RU, in that adequate staffing was associated with low RU. Staffing is, and has previously been, studied in relation to RU [24,54] and is included as a variable of interest in relation to RU in the Alberta Context Tool (ACT) [55]. Adequate staffing has previously been associated with higher RU and better patient outcomes [24]. The difference in results is difficult to reconcile but may reflect that optimal staffing for high-quality care is more complex than just counting the number of staff and assessing the skill mix [56]. Instead, according to Dubois and Singh [56], the focus should lie on staff skills and the effective use of those skills. Skill management is about optimizing the use of staff education, training, skills, knowledge, experience, and competence [56]. Here, this could imply that, although the number of staff was perceived adequate to meet patients’ needs, staff skills and competence regarding RU was not fully used, maybe even hindered by factors related to management. Such an interpretation might be linked to the association between less challenge and low RU, in which the experience of challenge includes a perception that an individual’s skills and knowledge are useful [45]. Managers that do not allow staff to fully make use of their skills and knowledge (e.g., regarding RU) might be a result of a narrowly defined management with misuse of staff competence. Further, we found that ambiguous role clarity (i.e., uncertainty about expectations and responsibilities, work goals, and objectives [45]), also hypothesized to be mediated by management, was linked to low RU. It seems reasonable that knowledge and clarity about what the nurse is expected to do, and why he or she is expected to do certain things, are a prerequisite for being able to reflect upon action and use of research findings.

The element to the very left in our analytic schedule representing Work Context is the “overarching” element, proposed to influence management within the context and, consequently, individual qualities and characteristics, psychological consequences for employees, and, ultimately, staff performance. In the final logistic regression model, the clinical setting turned out to be associated with RU in that nurses in psychiatric care rated their RU lower than nurses in hospital care. Associations between clinical specialty and RU were demonstrated in the systematic review by Squires et al. [16], where nurses in critical care areas scored higher on RU. Boström and colleagues [12] reported that nurses working in elderly care scored higher on items related to the application of EBPs. Research on nurses’ RU in psychiatric care, specifically research that compares RU among psychiatric nurses with nurses in other care settings, is to our knowledge rare. Bahtsevani et al. [57] reported both low use of evidence-based literature and limited access to the literature in Swedish psychiatric nurses. Koivunen et al. [58] found that, when using literature databases, nurses in psychiatric hospitals showed deficient information-retrieval skills. Similar to barriers to nurses’ RU [14], a recent study of barriers and facilitators to guideline implementation among healthcare personnel in psychiatric care [59] identified three major categories of barriers related to the organization, the individual, and the evidence. It is difficult to determine what specific characteristics of the psychiatric setting might hinder RU. A possible methodological explanation could be that the examples of each kind of RU included in each RU item in the survey were focused on somatic care interventions, which could have limited the psychiatric nurses in reflecting on their own practice, thereby contributing to the gap increase between ratings of RU in psychiatric care and other settings.

Among the sociodemographic characteristics, sex was associated with RU in the final model, (i.e., male nurses were more likely to rate low RU). Given our data, a clear explanation of that finding is impossible. Various gender differences in nursing are known from previous research; for instance, career preferences (e.g., preferred healthcare areas of work) tend to differ between male and female nurses [60,61]: male nurses more often have management positions [61] and work values differ (e.g., males value the salary and advancement possibilities as more important than do female nurses) [61,62]. Gender-based differences during education have also been reported [32,63]. These differences might indicate that men in our sample are overrepresented in positions or roles that, for some reason, lead to lower RU ratings. However, this relationship needs further investigation.

When it comes to factors related to undergraduate education, the LANE project offers a unique opportunity to study educational factors in a national sample and to connect aspects of education to later working life. Little is known about how educational factors predict future professional behavior. This has, to our knowledge, not been studied before in relation to RU. In competition with the variables representing the situation during the second year as a nurse, contribution to discussion in class was identified as a determining factor of RU in the final regression model. Whether this result is related to individuals’ differing approaches to education and the broad diversity of academic ability or varying quality of teaching and other characteristics of nursing schools cannot be concluded from these results. The fact that schools vary in their “quality” and extent of achievement of educational goals has been reported by the Swedish National Agency for Higher Education in its national review of nursing education [30]. Variable results in student-assessed educational outcomes between nursing schools were also shown in two other studies in the LANE project [31,32].

Nurses’ transition from education into working life is often described as stressful and overwhelming, which is proposed to be a response to the lack of a supportive environment [64-66]. The transition often brings about challenges concerning exercising EBPs [67]. This socialization process [68,69] that new nurses undergo to “become a nurse” and a member of the nursing staff is highly dependent on the work context. Such a dependency of the work context might explain the emphasis of variables related to Work Context, Management, and Individual Perceptions of Work, rather than educational factors in our results. Taken together, none of the variables representing Psychological Consequences for Employees were found to be associated with RU in the final regression model.

We found our analytic schedule helpful for both the data analysis and interpretation of our findings. The schedule also assisted in revealing the difficulty of separating organizational, individual, and educational factors. While the clinical setting can be considered as a clear organizational factor and sociodemographic characteristics as clearly individual, the other elements represent self-rated individual perceptions and reactions on work and educational setting. Self-ratings cause difficulties in keeping individual perceptions separate from the context that is to be described, which has to be considered when interpreting the results. Furthermore, the nature of several of the variables, such as work demands and challenges, makes it difficult to label them as distinctly individual or organizational. Contextual factors are individually interpreted, implying that perceptions and reactions on similar conditions might differ between individuals. This also indicates—depending on how variables are measured—that individual and organizational factors are interconnected.

### Methodological considerations

Despite not originally being developed to address RU, the modified NHS model used in this study includes some advantages that made us choose this model as an analytic schedule. For example, to some extent, it takes the interaction between the individual and the organizational/educational context into account. A number of challenges have been articulated in research on knowledge translation (e.g., increased attention to the role of the organizational context, including levels of context and interaction between levels) [70]. There is also a call for more sophisticated study designs and statistical techniques since correlational (bivariate) designs have been predominant so far, failing to account for the complex relationships between various factors and RU [70]. Through the use of our analytic schedule and multivariate approach to data analysis, we have attempted to meet some of these challenges. By the multivariate approach, we intended to manage overlaps between the variables, sorting out the ones having unique associations with the outcome. Multivariate modelling is a necessary approach in the investigation of factors associated with RU. In this respect, we found the schedule helpful as an organizing tool to guide the analysis. Although the analytic schedule was helpful, it had limitations. For instance, it has a quasi-causality in which feedback loops and opposite directions of associations are conceivable. Causal relationships have not been tested in the present study and the hypothesized “one-way linearity” from left to right implies that dynamics, like feedback loops, are not considered.

The LANE project brings much strength to this study, including its national approach, sample size, longitudinal design, and data from undergraduate education. Our sample included only individuals with complete responses to the RU variables, excluding nonresponders and participants who responded “Don’t know.” Even though this led to a reduced sample size, it was considered inevitable since imputation of missing data was considered not appropriate in the cluster analysis underlying the outcome variable. However, the sample representativeness on demographic and health-related variables, as well as cohort representativeness in the demographic variables tested, support a high generalizability of the findings to our population (i.e., newly graduated Swedish nurses).

It is possible that the different kinds of RU have partly different determinants. However, this possibility was not studied here since the outcome was overall low RU (i.e., low or very low use in all three kinds of RU). Independent variables were mainly dichotomized to facilitate interpretation. In the step 1 bivariate analysis, various ways to dichotomize were tested, without a change in the results, indicating the robustness of the results.

## Conclusions

This study provides new knowledge about the factors associated with low RU. Our findings suggest that several of the identified factors are potentially modifiable. In addition, the study provides knowledge that is important as a basis for designing future interventions to improve RU in clinical practice. The results illustrate the multifarious influences that play a role in RU, such as that both individual (including individual perceptions and management of education) and organizational factors are associated with RU. Our use of an analytic schedule and the multivariate approach to data analysis constitutes methodological strengths, useful for formulating hypotheses about how factors are related to each other and how they determine RU. In addition, the schedule assisted in illustrating the difficulties of designating variables as organizational, individual, or educational. Future research would benefit from a multivariate approach to avoid the separation of individual and organizational/contextual variables, thus better illustrating reality. Furthermore, an increased focus on educational factors is necessary to improve the possibility of making early interventions aiming to increase nurses’ RU.

## Competing interests

The authors declare that they have no competing interests.

## Authors’ contributions

All authors contributed to the conception and design of the study. PG, AE, and LW secured funding for the project. All authors were involved in data collection. Data analysis was performed by HF, AR, and PG. HF was responsible for the drafting of the manuscript. All authors made critical revisions to the manuscript for important intellectual content and approved the final version of the manuscript.

## Supplementary Material

Additional file 1 **Information about independent variables and how they were managed in the logistic regression analyses **[71]**,**[72]**.** Click here for file

Additional file 2 Model evaluation statistics from the final logistic regression model. Click here for file
