# Supplementary material for: Nurses’ research utilization two years after graduation—a national survey of associated individual, organizational, and educational factors
Source: Implement Sci. 2012 May 18;7:46. doi: 10.1186/1748-5908-7-46 (PMC3503782; doi:10.1186/1748-5908-7-46)
Supplement: Additional file 1 — Information about independent variables and how they were managed in the logistic regression analyses [71],[72]. [file 1748-5908-7-46-S1.doc]

**Additional file 1.** Independent variables included in the logistic regression analyses, including information about their origins, how response categories were managed and frequency distributions. * = reference categories. Bold headings in italics = elements. Headings in italics = sub-elements.

| **Variables included in the elements and sub-elements of the analytic schedule** | **Response categories** | **Total**  **n (%)** | **Low RU**  **n (%)** |
| --- | --- | --- | --- |
| ***Work context*** |  |  |  |
| Present form of employment | Temporary, employed by the hour, engaged in project, other non-permanent position*  Permanent | 350 (41.9)    485 (58.1) | 187 (53.4)    274 (56.5) |
| Clinical setting | Hospital care (acute somatic care)* | 621 (73.9) | 320 (51.5) |
|  | Primary care (community health care centres, home care) | 34 (4.0) | 20 (58.8) |
|  | Elder care (special housing for seniors) | 103 (12.2) | 55 (53.4) |
|  | Psychiatric care (hospitals and outpatient clinics) | 83 (9.9) | 66 (79.5) |
| Full- or part-time | ≤75%*  >75% | 131 (15.8)  699 (84.2) | 70 (53.4)  385 (55.1) |
| Work shift | Day, evening, night* | 623 (78.8) | 333 (53.5) |
|  | Monday to Friday (day, evening) | 102 (12.9) | 62 (60.8) |
|  | Night | 66 (8.3) | 33 (50.0) |
| ***Management*** |  |  |  |
| Work overtime | About once a week, about once a month, <once a month, never*  Several times per week | 668 (79.3)  174 (20.7) | 379 (56.7)  83 (47.7) |
| Adequate staffing compared with patients’ need of care | No*  Yes | 517 (61.9)  318 (38.1) | 272 (52.6)  188 (59.1) |
| Individual plan for competence development | Yes* | 287 (34.2) | 142 (49.5) |
|  | No, Don’t know | 551 (65.8) | 319 (57.9) |
| Experience of role clarity  Clarity of work goals and objectives, awareness of expectations and responsibility [45]  (3 items, Cronbach’s α = 0.75) | High (Very often/always, quite often)* | 607 (72.3) | 314 (51.7) |
|  | Low (Sometimes, quite seldom, very seldom/never) | 233 (27.7) | 148 (63.5) |
| Experience of leadership  Support from a superior, empowering leadership, fair leadership [45]  (6 items, Cronbach’s α = 0.91) | High (Very often/always, quite often)* | 244 (29.0) | 122 (50.0) |
|  | Low (Sometimes, quite seldom, very seldom/never) | 596 (71.0) | 340 (57.0) |

| **Variables included in the elements and**  **sub-elements of the analytic schedule** | **Response categories** | **Total**  **n (%)** | **Low RU**  **n (%)** |
| --- | --- | --- | --- |
| ***Individual qualities and characteristics*** |  |  |  |
| *Socio-demographic characteristics* |  |  |  |
| Sex | Women*  Men | 754 (89.2)  91 (10.8) | 400 (53.1)  64 (70.3) |
| Age | ≤ 30 years* | 368 (43.6) | 198 (53.8) |
|  | > 30 years | 477 (56.4) | 266 (55.8) |
| Previous assistant nurse training | Yes* | 386 (46.1) | 211 (54.7) |
|  | No | 451 (53.9) | 250 (55.4) |
| Further study after nursing degree  Specialist nurse, midwife, master’s degree, doctoral degree | No*  Have studied, study now | 703 (84.2)  132 (15.8) | 373 (53.1)  85 (64.4) |
| *Individual perceptions and management of education* |  |  |  |
| Global importance of studies:  1 (one of the most important things in life) to 7 (one of the least important things in life) [45] | Important (1-3)* | 557 (72.8) | 291 (52.2) |
|  | Less important (4-7) | 208 (27.2) | 130 (62.5) |
| Time allocated to studies [50,52] | Full-time* | 314 (41.3) | 177 (56.4) |
|  | >Full-time | 208 (27.4) | 97 (46.6) |
|  | 75% of full-time, 50% of full-time, <50% of full-time | 238 (31.3) | 143 (60.1) |
| Asked questions in class [50,52] | Often* | 157 (20.7) | 77 (49.0) |
|  | Sometimes, seldom, never | 603 (79.3) | 339 (56.2) |
| Contributed to discussions in class [50,52] | Often* | 186 (24.5) | 86 (46.2) |
|  | Sometimes, seldom, never | 574 (75.5) | 330 (57.5) |
| Quality during education, scientific theory and method | Very good, quite good* | 459 (61.7) | 249 (54.2) |
|  | Neither good nor bad, quite bad, very bad | 285 (38.3) | 161 (56.5) |
| Feel prepared to manage work as nurse:  1 (totally agree) to 7 (do not agree at all)  [71] | Yes (1-3)* | 471 (61.6) | 243 (51.6) |
|  | No (4-7) | 294 (38.4) | 177 (60.2) |
| *Individual perceptions of work, 2^nd^ year* |  |  |  |
| Job demands  Quantitative job demands (time pressure and amount of work), decision demands (demands for quick and complex decisions) [45]  (4 items, Cronbach’s α = 0.75) | Low (Sometimes, quite seldom, very seldom or never)*  High (Very often/always, quite often) | 568 (67.9)  268 (32.1) | 318 (56.0)  143 (53.4) |
|  |  |  |  |

| **Variables included in the elements and**  **sub-elements of the analytic schedule** | **Response categories** | **Total**  **n (%)** | **Low RU**  **n (%)** |
| --- | --- | --- | --- |
| Positive challenge at work  Perception that skills and knowledge are useful and that work is meaningful and positively challenging [45]  (3 items, Cronbach’s α = 0.70) | High (Very often/always, quite often)* | 684 (81.8) | 353 (51.6) |
|  | Low (Sometimes, quite seldom, very seldom or never) | 152 (18.2) | 108 (71.1) |
| Control  Influence on decisions in the work situation, control of work pacing [45]  (4 items, Cronbach’s α = 0.66) | High (Very often/always, pretty often)* | 65 (7.7) | 38 (58.5) |
|  | Low (Sometimes, quite seldom, very seldom or never) | 775 (92.3) | 424 (54.7) |
|  |  |  |  |
| ***Psychological consequences for employees*** |  |  |  |
| Often think about leaving the profession  1 (Completely accurate) to 5 (Completely inaccurate) [72] | No (3-5)*  Yes (1-2) | 740 (88.4)  97 (11.6) | 397 (53.6)  62 (63.9) |
| Disengagement  One of two core dimensions of burnout [48]. Distancing oneself from work, experiencing negative attitudes towards work.  (6 items, Cronbach’s α = 0.84)^1^ | Low (2.51–4.0)*  High (0-2.50) | 725 (86.5)  113 (13.5) | 390 (53.8)  73 (64.6) |
| Exhaustion  One of two core dimensions of burnout [48]. A consequence of intensive physical, affective and cognitive strain. (6 items, Cronbach’s α = 0.75) ^1^ | Low (2.51–4.0)*  High (0-2.50) | 562 (67.1)  276 (32.9) | 307 (54.6)  156 (56.5) |
| Mastery  Contentment with own quantity and quality of the work performed as well as with own ability to solve problems at work [45] (3 items, Cronbach’s α = 0.77) | High (Very often/ always, quite often)* | 570 (67.9) | 296 (51.9) |
|  | Low (Sometimes, quite seldom, very seldom or never) | 270 (32.1) | 166 (61.5) |

^1^ Exhaustion and disengagement scales from the Oldenburg Burnout Inventory measuring professional burnout [48]. Response scales: 1 (‘Completely accurate), 2 (‘Quite accurate’), 3 (‘Not very accurate’), 4 (‘Completely inaccurate’).
