# Supplementary material for: Nurses’ research utilization two years after graduation—a national survey of associated individual, organizational, and educational factors
Source: Implement Sci. 2012 May 18;7:46. doi: 10.1186/1748-5908-7-46 (PMC3503782; doi:10.1186/1748-5908-7-46)
Supplement: Additional file 2 — Model evaluation statistics from the final logistic regression model. [file 1748-5908-7-46-S2.doc]

**Additional file 2.** Model evaluation statistics for the final logistic regression model (Step 3). Variables (in italics) significantly associated with low RU within each element/sub-element (Step 2) were entered sequentially according to the analytic schedule.

|  | **-2LL^*^** | **Delta Chi square** | **Hosmer-Lemeshow** |
| --- | --- | --- | --- |
| Work context/ *Clinical setting* | 987.2 | 25.3, df=3, p=0.001 | 0.001, df=1, p=1.000 |
| Management/ *Staffing, role clarity* | 974.9 | 12.3, df=2, p=0.002 | 2.1, df=4, p=0.721 |
| Socio-demographic characteristics  and Individual perceptions and  management of education /  *Sex, further training, student activity* | 956.2 | 18.7, df=3, p=0.001 | 2.2, df=7, p=0.951 |
| Individual perceptions of work/  *Challenge* | 943.2 | 13.0, df=1, p=0.001 | 1.9, df=8, p=0.984 |
| Psychological consequences for  employees/ *Mastery* | 941.2 | 2.08, df=1, p=0.149 | 2.8, df=8, p=0.944 |
|  |  | **Model Chi square:**  71.3, df=10, p=0.001 |  |

^*^ -2 log likelihood
